# Combined analysis of three genome-wide association studies on vWF and FVIII plasma levels

**DOI:** 10.1186/1471-2350-12-102

**Published:** 2011-08-02

**Authors:** Guillemette Antoni, Tiphaine Oudot-Mellakh, Apostolos Dimitromanolakis, Marine Germain, William Cohen, Philip Wells, Mark Lathrop, France Gagnon, Pierre-Emmanuel Morange, David-Alexandre Tregouet

**Affiliations:** 1 UMR_S 937, INSERM, Boulevard de l'Hopital, Paris, 75013, France; 2 UMR_S 937, ICAN Institute, Université Pierre et Marie Curie, Boulevard de l'Hopital, 75013, Paris, France; 3 Dalla Lana School of Public Health, University of Toronto, College Street, Toronto, M5T 3M7, Ontario, Canada; 4 UMR_S 626, INSERM, rue Saint-Pierre, Marseille, 13385, France; 5 UMR_S 626, Université de la Méditerranée, rue Saint-Pierre, Marseille, 13385 France; 6 Department of Medicine, Ottawa Hopital Research Institute, Carling Avenue, Ottawa, K1Y 4E9, Ontario, Canada; 7 Institut de Génomique, Centre National de Génotypage, Commissariat à l'Energie Atomique, rue Gaston Crémieux, Evry, 91057, France

## Abstract

**Background:**

Elevated levels of factor VIII (FVIII) and von Willebrand Factor (vWF) are well-established risk factors for cardiovascular diseases, in particular venous thrombosis. Although high, the heritability of these traits is poorly explained by the genetic factors known so far. The aim of this work was to identify novel single nucleotide polymorphisms (SNPs) that could influence the variability of these traits.

**Methods:**

Three independent genome-wide association studies for vWF plasma levels and FVIII activity were conducted and their results were combined into a meta-analysis totalling 1,624 subjects.

**Results:**

No single nucleotide polymorphism (SNP) reached the study-wide significance level of 1.12 × 10^-7 ^that corresponds to the Bonferroni correction for the number of tested SNPs. Nevertheless, the recently discovered association of *STXBP5*, *STX2*, *TC2N *and *CLEC4M *genes with vWF levels and that of *SCARA5 *and STAB2 genes with FVIII levels were confirmed in this meta-analysis. Besides, among the fifteen novel SNPs showing promising association at p < 10^-5 ^with either vWF or FVIII levels in the meta-analysis, one located in *ACCN1 *gene also showed weak association (*P *= 0.0056) with venous thrombosis in a sample of 1,946 cases and 1,228 controls.

**Conclusions:**

This study has generated new knowledge on genomic regions deserving further investigations in the search for genetic factors influencing vWF and FVIII plasma levels, some potentially implicated in VT, as well as providing some supporting evidence of previously identified genes.

## Background

Elevated plasma levels of factor VIII (FVIII) and von Willebrand factor (vWF), two key molecules of the coagulation cascade, are well-established risk factors for venous thrombosis (VT) [1-3]. More recent evidence shows that these plasma hemostatic proteins are also risk factors for other cardiovascular diseases (CVD) [4-8]. The broader role of FVIII and vWF is further supported by studies showing that genetic factors modulating the variability of these proteins are also associated with CVD. These include single nucleotide polymorphisms (SNPs) at the *BAI3 *[9], *LDLR *[5,10], *VWF *[4] and *ABO *[11] genes, the latter being associated with other quantitative risk factors for CVD [12,13].

The estimated heritability of FVIII and vWF levels range between 40% and 60% [14,15] among which about 20% is attributable to the *ABO *locus. A genome wide association study (GWAS) within the CHARGE consortium [16] has recently identified five new genes, apart from their structural genes and *ABO*, consistently influencing vWF and/or FVIII plasma levels. These include *CLEC4M*, *SCARA5*, *STX2*, *STXBP5 *and *TC2N*, collectively explaining ~10% of the variability of each two traits. These observations suggest that there are additional genetic factors remaining to be identified and contributing to the hidden heritability of these quantitative traits.

The increased power of selected samples has long been recognized in family-based studies but more recently the putative advantages of carefully selected samples for quantitative trait analysis of unrelated subjects has also been highlighted [17]. Therefore, we undertook the combined analysis of individual data from three GWAS performed in samples of VT patients and in extended families ascertained on VT and Factor V Leiden (FVL) to identify novel genetic factors implicated in the variation of plasma levels of FVIII and vWF.

## Methods

### Overall strategy

To achieve our primary goal of identifying new genetic factors that could influence vWF and/or FVIII plasma levels, we used data from three carefully selected independent GWAS. Great attention was drawn to the homogeneity across samples in terms of - ethnic background (most individuals were of French origin), - exclusion criteria with respect to rare forms of inherited thrombophilia, - objectively diagnosed VT, - studied intermediate phenotypes (although some adjustments were done) and similar genotyping technologies (Illumina platform).

In the context of quantitative trait GWAS, individual genetic effect sizes are known to be small [18] and it is expected that a number of real associations do not reach genome-wide significance. Therefore, as part of our analytic strategy, we first tested for association in the individual studies, and results observed across samples were combined into a meta-analysis. We then focused on the consistency of associations across studies as our hypothesis was that real associations would more likely be consistently observed across studies given that each study samples were quite homogeneous with respect to the above-mentioned characteristics. Previously reported associations were also investigated using the above strategy.

As genetic variants associated to plasma levels of FVIII and vWF could be risk factors for VT, our secondary goal was to test the identified SNPs with VT using an *in silico *GWAS [19]. Analytic approaches and samples characteristics of the FVIII and vWF GWAS are described below.

### FVL-families sample

Five extended French-Canadian families were ascertained through single probands with idiopathic VT diagnosed at the Thrombosis Clinic of the Ottawa Hospital, and carrying the FVL mutation. VT cases secondary to cancer as well as rare forms of inherited VT (protein S, protein C, AntiThrombin deficiencies) were excluded. A pedigree was drawn from interviews with each potential probands. The largest families were invited to participate in the study - the family size and willingness to participate being the only criteria for the selection of the families (see Additional File 1, File S1 for the used questionnaire). The total number of family members was 255. Description of the extended families has been published elsewhere [9].

### MARTHA samples

The MARseille THrombosis Association (MARTHA) project is composed of two independent samples of VT patients, named MARTHA08 (N = 1,006) and MARTHA10 (N = 586). MARTHA subjects are unrelated caucasians consecutively recruited at the Thrombophilia center of La Timone hospital (Marseille, France) between January 1994 and October 2005. All patients had a documented history of VT and free of well characterized genetic risk factors including AT, PC, or PS deficiency, homozygosity for FV Leiden or FII 20210A, and lupus anticoagulant. They were interviewed by a physician on their medical history, which emphasized manifestations of deep vein thrombosis and pulmonary embolism using a standardized questionnaire (see Additional file 2, File S2). The thrombotic events were confirmed by venography, Doppler ultrasound, spiral computed tomographic scanning angiography, and/or ventilation/perfusion lung scan. All the subjects were of European origin, with the majority being of French descent.

The main characteristics of the three samples are shown in Table 1.

**Table 1:** Main Characteristics of the Studied Samples

	FVL Families N = 253	MARTHA08 N = 972	MARTHA10 N = 570
Age (SD)	40.4 (17.9)	45.7 (14.9)	49.2 (15.7)
Sex (% female)	50.6%	70.8%	58.2%
Smoking (%)	24.4%	24.9%	22.71%
History of VT (%)	5.95%	100%	100%
PT G20210A carriers	0.40%	15.9%	10.6%
FV Leiden carriers	24.9%	26.6%	14.1%
ABO blood group (%)			
O	40.6%	22.9%	22.4%
A	57.8%	61.8%	59.3%
B	1.6%	10.3%	14.4%
AB	-	5%	3.9%
FVIII (SD) IU/dL	118.6 (38.51)	138.70 (55.34)	130.2 (46.35)
vWF (SD) IU/dL	130.3 (53.24)	152.33 (68.23)	152.9 (63.93)

### In silico GWAS study on VT

In a previously published GWAS on VT [19], 419 early age of onset and the idiopathic character of VT (ie without environemental risk factors) (< 50 years) VT cases were compared to 1,228 healthy controls at 291,872 SNPs. Cases were patients from four different French medical centers (Grenoble, Marseille, Montpellier, Paris) selected according to the same criteria as the MARTHA samples, except with the restriction on age of onset. Controls were French subjects selected from the SUVIMAX population [20].

### Measurements

In the French-Canadian (FVL) sample, plasma levels of FVIII activity were measured by a clotting assay on the BCS instrument (Siemens Diagnostics, Marburg Germany) and vWF antigen was measured with a commercially available ELISA kit from Diagnostica Stago. The interassay coefficients of variation for FVIII were ~ 1% and 6.1% for vWF.

In MARTHA subjects, plasma coagulant activity and vWF antigen were assayed in an automated coagulometer (STA-R; Diagnostica Stago, Asnières, France). The interassay coefficients of variation for FVIII and vWF were 6.96% and 2.27% respectively.

### Genotyping

The French-Canadian sample was genotyped with the Illumina 660W-Quad Beadchip. The raw datafile contained data for 547,886 autosomal SNPs genotyped on 255 individuals. From these SNPs, 490,083 passed the quality control (QC) criteria of genotyping rate > 90% and more than 20 observations of the minor allele among all individuals. After removing the 88,390 SNPs that failed QC, the overall genotyping rate was 99.88%. The maximum missing rate per sample for all the 255 samples was 3.9%, with an average missing rate of 0.13%. The family structures had previously been checked using 1079 microsatellite markers and RELPAIR [9]. To further verify the correctness of the family structure, we used PREST [21] and computed IBD estimates for all the sample pairs, within and across pedigrees. PREST reported 14,949 Mendelian errors, which is equivalent to a very low Mendelian error rate of 0.012% among all genotypes. Genotypes showing Mendelian inconsistencies were excluded from the analysis. Finally, phenotypic and genotypic data were available on a total of 253 individuals.

The MARTHA08 study sample was typed in 2008 with the Illumina Human610-Quad Beadchip containing 567,589 autosomal SNPs while the MARTHA10 sample was recently typed (beginning of 2010) with the same Illumina Human660W-Quad Beadchip as in the FVL study sample. SNPs showing significant (P < 10^-5^) deviation from Hardy-Weinberg equilibrium, with minor allele frequency (MAF) less than 1% or genotyping call rate < 99%, in each study were filtered out. Individuals with genotyping success rates less than 95% were excluded from the analyses, as well as individuals demonstrating close relatedness as detected by pairwise clustering of identity by state distances (IBS) and multi-dimensional scaling (MDS) implemented in PLINK software [22]. Non-European ancestry was also investigated using the Eigenstrat program [23] leading to the final selection of 972 and 570 patients left for analysis in MARTHA08 and MARTH10, respectively. Plasma vWF levels were available in 834 and 537 MARTHA08 and MARTHA10 patients, respectively; corresponding numbers were 541 and 548 for plasma FVIII levels. A total of 442,728 SNPs were common to the three GWAS datasets (see Additional file 3, Figure S1).

### Statistical analysis

In the FVL families, association of SNPs with vWF and FVIII levels was tested by means of measured genotype linear association analysis as implemented in the SOLAR (version 4.0, http://solar.txbiomedgenetics.org/download.html) program. In MARTHA subjects, association was tested using linear model as implemented in the PLINK program [22].

**Figure 1:**
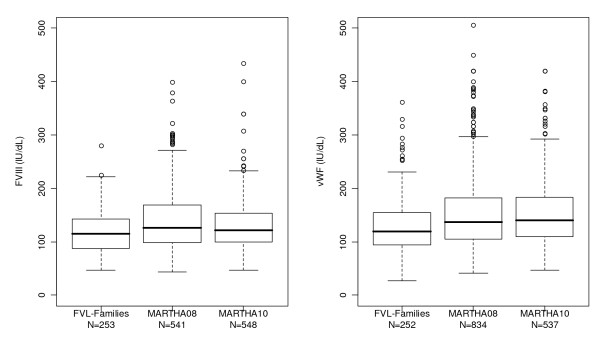
**Box Plot Distribution of FVIII (left) and vWF (right) Plasma Levels in the Three GWAS Datasets**.

In order to handle differences in phenotype distributions across studies (Figure 1), and any possible deviation from normality, plasma levels of vWF and FVIII were first normalized before any statistical analysis using the normal quantile transformation [24], separately in the French-Canadian sample, MARTHA08 and MARTHA10. This transformation assigns to each observed measurement the quantile value of the standard normal distribution that corresponds to the rank of this measurement in the original untransformed distribution. Transformed variables are then normally distributed making linear models applicable, and linear regression coefficients comparable across studies. Association analyses were then carried out on the transformed variables assuming additive allele effects (0,1, 2 coding according to the number of minor alleles), and adjusting for age, sex and *ABO *blood group as tagged by the *ABO *rs8176746, rs8176704 and rs505922 [19]. When appropriate, haplotype association analyses were carried out in MARTHA samples using THESIAS software [25] to handle the correlation between SNPs, that is linkage disequilibrium (LD). This widely used software implements a stochastic-EM algorithm that simultaneously estimates the frequencies and the effect on the studied phenotype of each inferred haplotype. Haplotype - phenotype associations are then assessed by means of likelihood ratio tests.

Results obtained in each GWAS datasets were combined in a meta-analysis using the GWAMA program [26]http://www.sph.umich.edu/csg/abecasis/metal. Both fixed-effect and random-effect models- based analyses were conducted. Regression coefficients characterizing the minor allele effect of each SNP were then combined (after having checked that the minor allele was the same in the different populations) using the inverse-variance method to provide an overall allelic estimate. All reported P values were 2-sided.

## Results

**Figure 2:**
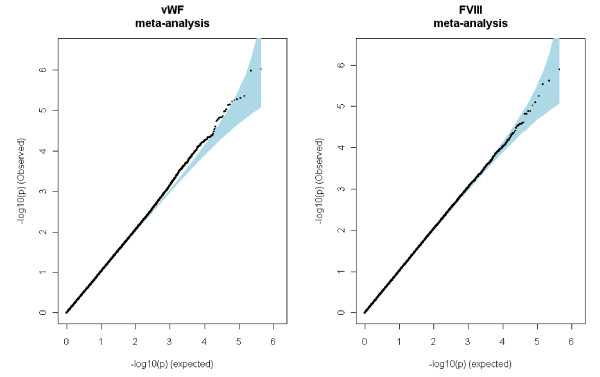
**Quantile-Quantile Plots of the Association Results from the Meta-Analysis of the Three GWAS Datasets**.

A total of 442,728 QC-validated SNPs were common to the three GWAS and were tested through a meta-analysis for association with vWF and FVIII plasma levels. Quantile-quantile plots did not reveal any inflation from what was expected under the null hypothesis of no association (Figure 2), and no SNP reached the study-wide significance level of 1.12 × 10^-7 ^that corresponds to the Bonferroni correction for the number of tested SNPs. Applying the less stringent Sidak correction corresponding to a significant threshold of p = 1.16 × 10^-7 ^would not have modified this conclusion. We then further focused on genetic effects that were consistent across studies and with combined p-value of less than 10^-5^. As fixed-effect and random-effect analyses provided similar results for most of the main associations (Tables 2 &3), the following discussion is based on results obtained from the fixed-effect model analysis.

**Table 2:** Ten SNPs Showing Association with vWF levels Across the Three GWAS Datasets With Combined Significance P-value < 10^-5^

Gene	SNP	Alleles*		**MAF**^**+**^	β (SE)	p	**I**^**2**^	**p**_**het**_	Random Effect		Fixed Effect	
									β (SE)	p	β (SE)	p
			FVL	0.47	-0.16 (0.08)	0.044						
*VPS8*	rs4686760	A/G	Martha08	0.46	-0.18 (0.04)	4.11 10^-5^	0	0.549	0.15 (0.03)	1.10 10^-6^	-0.15 (0.03)	1.08 10^-6^
			Martha10	0.45	-0.11 (0.05)	0.047						

			FVL	0.15	0.44 (0.11)	3.08 10^-4^						
	rs13361927	*G/A*	Martha08	0.06	0.28 (0.09)	0.003	0.53	0.119	-0.28 (0.09)	0.002	0.28 (0.06)	4.51 10^-6^
*EPB41L4A*			Martha10	0.05	0.11 (0.11)	0.316						
	
			FVL	0.12	0.46 (0.12)	8.35 10^-4^						
	rs379440	A/G	Martha08	0.04	0.31 (0.11)	0.004	0	0.502	-0.34 (0.07)	9.99 10^-7^	0.34 (0.07)	9.82 10^-7^
			Martha10	0.03	0.25 (0.14)	0.071						

			FVL	0.04	-0.01 (0.21)	0.977						
*ANKRD6*	rs6454764	*C/T*	Martha08	0.06	0.24 (0.09)	0.007	0.70	0.036	-0.29 (0.14)	0.035	0.31 (0.07)	5.12 10^-6^
			Martha10	0.05	0.54 (0.12)	8.97 10^-6^						

			FVL	0.27	0.34 (0.09)	2.82 10^-4^						
*KRT18P24*	rs1757948	*T/G*	Martha08	0.27	0.1 (0.05)	0.030	0.62	0.071	-0.18 (0.06)	0.003	0.15 (0.03)	7.37 10^-6^
			Martha10	0.30	0.15 (0.06)	0.009						

			FVL	0.19	0.15 (0.1)	0.127						
	rs1438993	G/A	Martha08	0.28	0.18 (0.05)	1.11 10^-4^	0	0.666	-0.16 (0.03)	6.34 10^-6^	0.16 (0.03)	6.25 10^-6^
			Martha10	0.27	0.12 (0.06)	0.052						
	
			FVL	0.20	0.19 (0.1)	0.062						
*desert*	rs10745527	T/G	Martha08	0.28	0.18 (0.05)	1.63 10^-4^	0	0.663	-0.16 (0.03)	5.51 10^-6^	0.16 (0.03)	5.43 10^-6^
			Martha10	0.27	0.11 (0.06)	0.056						
	
			FVL	0.18	0.17 (0.11)	0.098						
	rs2579103	T/G	Martha08	0.26	0.19 (0.05)	8.24 10^-5^	0	0.533	-0.16 (0.04)	7.72 10^-6^	0.16 (0.04)	7.61 10^-6^
			Martha10	0.25	0.1 (0.06)	0.090						

			FVL	0.04	-0.02 (0.19)	0.905						
*CDH2*	rs2298574	A/G	Martha08	0.08	-0.34 (0.08)	2.77 10^-5^	0.19	0.290	0.26 (0.07)	1.81 10^-4^	-0.27 (0.06)	5.67 10^-6^
			Martha10	0.07	-0.24 (0.1)	0.022						

			FVL	0.05	0.32 (0.18)	0.080						
*SAFB2*	rs732505	G/A	Martha08	0.09	0.24 (0.08)	0.001	0	0.929	-0.25 (0.06)	9.50 10^-6^	0.25 (0.06)	9.38 10^-6^
			Martha10	0.08	0.25 (0.1)	0.013						

*Common/rare alleles

^+ ^Allele frequency of the minor allele

**Table 3:** Six SNPs Showing Association with FVIII Activity Across the Three GWAS Datasets With Combined Significance P-value < 10^-5^

Gene	SNP	Alleles*		**MAF**^**+**^	β (SE)	p	**I**^**2**^	**p**_**het**_	Random Effect		Fixed Effect	
									β (SE)	p	β (SE)	p
			FVL	0.41	-0.12 (0.09)	0.156						
*LBH*	rs6708166	G/A	Martha08	0.40	-0.23 (0.06)	8.98e-05	0	0.478	-0.17 (0.04)	1.32 10^-6^	-0.17 (0.04)	1.30 10^-6^
			Martha10	0.42	-0.15 (0.05)	0.007						

			FVL	0.42	-0.20 (0.08)	0.014						
*FAM46A*	rs1321761	T/C	Martha08	0.45	-0.10 (0.06)	0.074	0	0.451	-0.15 (0.04)	9.67 10^-6^	-0.15 (0.04)	9.54 10^-6^
			Martha10	0.47	-0.19 (0.05)	5.93e-04						

			FVL	0.17	0.28 (0.11)	0.012						
*VAV2*	rs12344583	A/G	Martha08	0.20	0.19 (0.07)	0.006	0	0.716	0.20 (0.04)	8.03 10^-6^	0.20 (0.04)	7.92 10^-6^
			Martha10	0.18	0.17 (0.07)	0.012						

			FVL	0.16	0.52 (0.12)	1.36e-05						
*S*TAB2	rs7306642	*C/A*	Martha08	0.07	0.22 (0.11)	0.057	0.59	0.086	0.31 (0.10)	0.002	0.30 (0.06)	2.95 10^-6^
			Martha10	0.07	0.20 (0.1)	0.052						

			FVL	0.53	0.09 (0.08)	0.293						
	rs1354492	*G/A*	Martha08	0.49	0.23 (0.05)	1.20e-05	0.39	0.192	0.16 (0.04)	5.47 10^-6^	0.16 (0.03)	2.41 10^-6^
*ACCN1*			Martha10	0.47	0.12 (0.05)	0.027						
	
			FVL	0.22	-0.29 (0.1)	0.004						
	rs12941510	G/A	Martha08	0.31	-0.17 (0.06)	0.002	0.12	0.321	-0.17 (0.04)	2.18 10^-5^	-0.17 (0.04)	5.67 10^-6^
			Martha10	0.33	-0.12 (0.06)	0.029						

*Common/rare alleles

^+ ^Allele frequency of the minor allele

**Figure 3:**
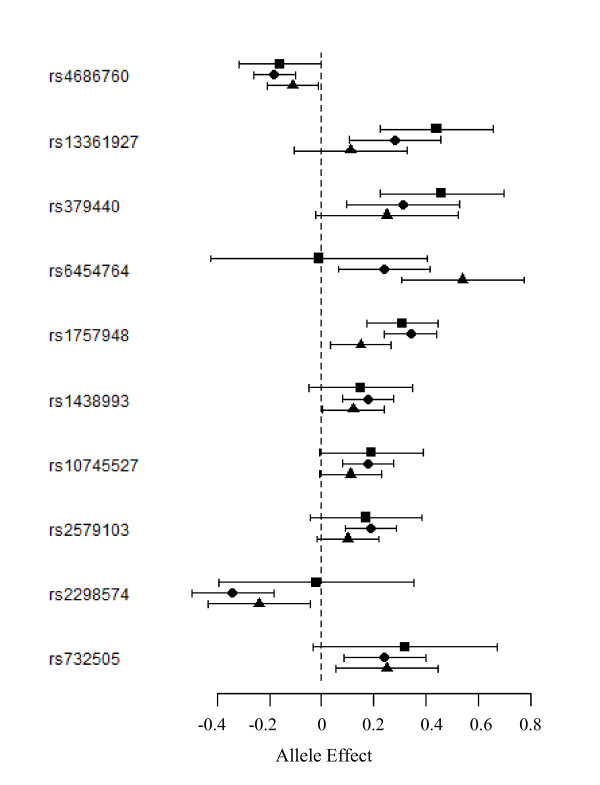
**Forest plot representation of the ten SNPs that associated the most with vWF levels in the Three GWAS Datasets**. Results observed in the FVL families, MARTHA08 and MARTHA10 studies are depicted by square, circle and triangle, respectively.

Ten SNPs covering seven different genes (Figure 3 - Table 2) were associated with plasma vWF levels at p < 10^-5 ^with no strong evidence for heterogeneity across GWAS as the lowest Mantel-Haenszel observed p-value, p = 0.036, for the ANKDR6 rs645764 would not pass multiple testing correction for testing ten SNPs. The strongest association was observed for rs379440 (*P *= 9.82 10^-6^) mapping the *EPB41L4A *gene (Table 2). Another SNP at this locus was also associated with vWF, rs13361927 (*P *= 4.51 10^-6^), but its association was due to its complete LD with rs379440, with pairwise r^2 ^of 0.78, 0.69 and 0.62 in FVL, MARTHA08 and MARTHA10, respectively. Other vWF-associated SNPs included the *SAFB2 *rs732505 (*P *= 9.38 10^-6^), *VPS8 *rs4686760 (*P *= 1.08 10^-6^) and the *KRT18P24 *rs1757948 (P = 7.37 10^-6^). The last three SNPs, rs1438993, rs10745527, rs2579103 (with P~ 6 10^-6^), were located at the 12q21.33 locus with no known mapped gene and were in nearly complete association. Altogether, the independent signals derived from the rs4686760, rs379440, rs1757948, rs10745527 and rs732505 explained up to 5.7% and 3.8% of the variability of plasma vWF levels in MARTHA08 and MARTHA10, respectively, and 5.3% in the pooled MARTHA samples.

**Figure 4:**
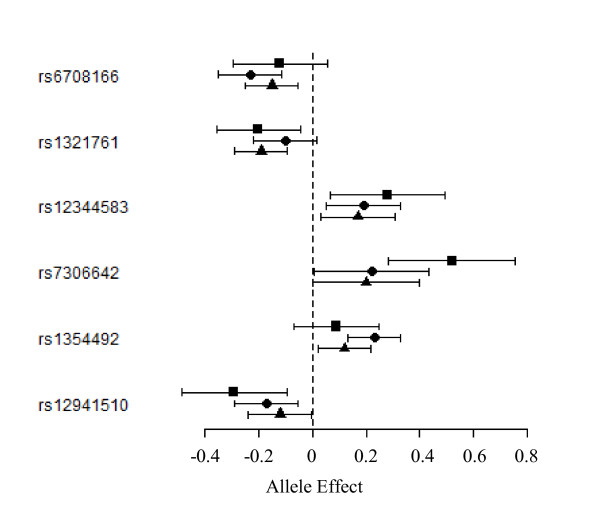
**Forest plot representation of the six SNPs that associated the most with FVIII levels in the Three GWAS Datasets**. Results observed in the FVL families, MARTHA08 and MARTHA10 studies are depicted by square, circle and triangle, respectively.

None of the ten vWF-associated SNPs were associated with plasma FVIII levels (all p > 0.05). However, six additional SNPs were specifically associated to FVIII levels with homogeneous effects (Mantel-Haenszel p-value > 0.05) across studies (Figure 4 - Table 3). The strongest effect (P = 2.95 10^-6^) was observed for rs7306642, a non synonymous Pro2039Thr variant within the *S*TAB2 gene, which was one of the recently identified genes by the CHARGE consortium. However, our hit rs7306642 was not in LD with any of the two *S*TAB2 SNPs recently identified, rs4981022 (r^2 ^< 0.01 in the three studies) and rs4981021 that served as a proxy for rs12229292 (r^2 ^< 0.07 in the three studies). Other FVIII-associated SNPs included the rs6708166 (*P *= 1.30 10^-6 ^) in the proximity of *LBH*, the rs1321761 ~ 300 kb apart from *FAM46A *(*P *= 9.54 10^-6 ^) and the intronic *VAV2 *rs12344583 (*P *= 7.92 10^-6 ^) (Table 3). Lastly, two SNPs within the *ACCN1 *gene, rs1354492 and rs12941510, were found modulating FVIII plasma levels, the A allele of the former being associated with increased FVIII levels (β = +0.16, *P *= 2.42 10^-6^) and the A allele of the latter being associated with decreased levels (β = -0.17, *P *= 5.67 10^-6^). These two SNPs were in complete negative LD generating three haplotypes, the sole carrying the rs1354492-A allele being associated with highest levels (see Additional file 4, Table S1). Altogether, these five SNPs (i.e. rs6708166, rs1321761, rs12344583, rs7306642, rs1354492) explained 8.2% and 4.6% of the variability of FVIII levels in MARTHA08 and MARTHA10, respectively, and 6.3% in the combined MARTHA samples.

We then used our GWAS datasets to investigate SNPs that had previously been reported associated with vWF and/or FVIII [4,5,9,16]. As shown in Supplementary Table two, marginal associations (P < 0.05) with vWF levels at *STXBP5*, *VWF*, *STX2*, *TC2N *and *CLEC4M *were also observed in our study, the strongest (P = 1.3 10^-4^) being for SNP rs216335 at the structural *VWF *gene. All these associations were consistent (i.e the same allele was associated with a genetic effect in the same direction on the studied phenotype) with those previously reported. Together, these associations explained an additional 1.4% and 3.2% of the variance of plasma levels of vWF in MARTHA08 and MARTHA10, respectively. We did not observe any evidence for an effect of *S*TAB2 rs4981022 or *BAI3 *rs9363864, while the effect of *SCARA5 *rs2726953 was heterogeneous across the studies. For FVIII levels, we observed marginal associations of *SCARA5 *rs9644133 (P = 0.009) and *VWF *rs1063856 (P = 0.020) that were consistent with those previously reported (Table 4), these two SNPs explaining 0.7% and 0.2% of FVIII variability in MARTHA08 and MARTHA10, respectively. No trend for association was observed for the previously reported associations with *STXBP5*, *S*TAB2 nor *LDLR *SNPs (Table 5).

**Table 4:** Association of Previously Identified SNPs with vWF Levels in the three GWAS Datasets

Gene	SNP	Alleles*		**MAF**^**+**^	β (SE)	p	**I**^**2**^	**p**_**het**_	Random Effect		Fixed Effect	
									β (SE)	p	β (SE)	p
			FVL	0.42	0.04 (0.08)	0.618						
*BAI3*	rs9363864	A/G	Martha08	0.52	0.03 (0.04)	0.421	0	0.838	0.02 (0.03)	0.461	0.02 (0.03)	0.461
			Martha10	0.49	-0.002 (0.05)	0.973						

			FVL	0.43	-0.08 (0.08)	0.366						
*STXBP5*	rs9390459	G/A	Martha08	0.42	-0.06 (0.04)	0.197	0	0.545	-0.09 (0.03)	0.005	-0.09 (0.03)	0.005
			Martha10	0.43	-0.13 (0.05)	0.011						

			FVL	0.20	-0.08 (0.10)	0.446						
*SCARA5*	rs10866867^(1)^	G/T	Martha08	0.25	0.17 (0.05)	4.88e-04	0.71	0.03	0.05 (0.07)	0.466	0.09 (0.04)	0.015
			Martha10	0.25	0.01 (0.06)	0.830						

			FVL	0.06	-0.28 (0.19)	0.141						
	rs216335^(2)^	G/A	Martha08	0.08	-0.23 (0.08)	0.003	0	0.945	-0.23 (0.06)	1.31 10^-4^	-0.23 (0.06)	1.30 10^-4^
			Martha10	0.06	-0.21 (0.11)	0.059						
	
			FVL	0.45	0.07 (0.08)	0.371						
*VWF*	rs1063856^(3)^	A/G	Martha08	0.37	0.08 (0.05)	0.094	0	0.889	0.09 (0.03)	0.006	0.09 (0.03)	0.006
			Martha10	0.38	0.11 (0.05)	0.041						
	
			FVL	0.48	-0.04 (0.08)	0.612						
	rs7306706	A/G	Martha08	0.45	0.02 (0.04)	0.634	0	0.754	0.01 (0.03)	0.664	0.01 (0.03)	0.664
			Martha10	0.46	0.03 (0.05)	0.604						

			FVL	0.30	-0.05 (0.09)	0.601						
*S*TAB2	rs4981022	T/C	Martha08	0.30	0.02 (0.05)	0.652	0	0.541	-0.01 (0.03)	0.664	-0.01 (0.03)	0.664
			Martha10	0.28	-0.06 (0.06)	0.333						

			FVL	0.33	0.01 (0.09)	0.863						
*STX2*	rs4334059^(4)^	C/T	Martha08	0.37	0.08 (0.04)	0.067	0.01	0.363	0.1 (0.03)	0.004	0.1 (0.03)	0.003
			Martha10	0.36	0.15 (0.06)	0.008						

			FVL	0.52	0.05 (0.08)	0.548						
*TC2N*	rs2402074^(5)^	G/A	Martha08	0.48	0.04 (0.04)	0.382	0	0.509	0.07 (0.03)	0.033	0.07 (0.03)	0.033
			Martha10	0.47	0.12 (0.05)	0.030						

			FVL	0.22	-0.07 (0.1)	0.515						
*CLEC4M*	rs868875	A/G	Martha08	0.32	-0.10 (0.05)	0.036	0	0.762	-0.08 (0.03)	0.026	-0.08 (0.03)	0.026
			Martha10	0.35	-0.05 (0.06)	0.424						

* Common/rare alleles

^+ ^Allele frequency of the minor allele

^(1) ^rs10866867 serves as proxy for rs2726953 (r^2 ^= 0.92); ^(2) ^rs216335 serves as proxy for rs216318 (r^2 ^= 1)

^(3) ^rs1063856 serves as proxy for Rs1063857 (r^2 ^= 1); ^(4) ^rs4334059 serves as proxy for rs7978987 (r^2 ^= 1.0

^(5) ^rs2402074 serves as proxy for rs10133762 (r^2 ^= 0.96); No good proxy with r^2 ^> 0.5 was available for the *VWF *rs4764478

**Table 5:** Association of Previously Identified SNPs with FVIII Activity in the three GWAS Datasets

Gene	SNP	Alleles*		**MAF**^**+**^	β (SE)	p	**I**^**2**^	**p**_**het**_	Random Effect		Fixed Effect	
									β (SE)	p	β (SE)	p
			FVL	0.43	0.15 (0.08)	0.083						
*STXBP5*	rs9390459	*G/A*	Martha08	0.42	-0.08 (0.06)	0.158	0.65	0.059	-0.02 (0.06)	0.795	-0.04 (0.03)	0.310
			Martha10	0.43	-0.07 (0.05)	0.199						

			FVL	0.24	-0.08 (0.1)	0.433						
*SCARA5*	rs9644133	C/T	Martha08	0.17	-0.16 (0.07)	0.029	0	0.753	-0.12 (0.05)	0.009	-0.12 (0.05)	0.009
			Martha10	0.18	-0.10 (0.07)	0.152						

			FVL	0.45	0.11 (0.08)	0.170						
*VWF*	rs1063856	A/G	Martha08	0.37	0.09 (0.06)	0.114	0	0.843	0.08 (0.03)	0.020	0.08 (0.03)	0.020
			Martha10	0.38	0.06 (0.05)	0.249						

			FVL	0.27	-0.13 (0.09)	0.146						
*S*TAB2	rs4981021^(1)^	G/A	Martha08	0.32	-0.02 (0.06)	0.737	0	0.389	-0.02 (0.04)	0.521	-0.02 (0.04)	0.521
			Martha10	0.29	0.02 (0.06)	0.782						

			FVL	0.14	-0.03 (0.11)	0.816						
	rs2228671	C/T	Martha08	0.11	0.11 (0.09)	0.193	0.46	0.157	-0.01 (0.07)	0.890	-0.01 (0.05)	0.894
*LDLR*			Martha10	0.10	-0.13 (0.09)	0.161						
	
			FVL	0.38	-0.25 (0.09)	0.005						
	rs688	C/T	Martha08	0.45	0.06 (0.05)	0.235	0.79	0.010	-0.05 (0.08)	0.531	-0.02 (0.03)	0.652
			Martha10	0.45	-0.007 (0.05)	0.901						

* Common/rare alleles

^+ ^Allele frequency of the minor allele

^(1) ^rs4981021 serves as proxy for rs12229292 (r^2 ^= 0.88)

We have recently observed that, among the newly identified vWF and/or FVIII genes by the CHARGE consortium, *TC2N *could also be associated with VT risk [27]. Therefore we investigated the effect of the SNPs identified in our meta-analysis on the risk of VT. Our working hypothesis was that SNPs associated with increased (decreased, resp.) plasma levels of these two molecules could be associated with increased (decreased, resp.) risk of disease. For this, we used the results of our previously published GWAS based on 419 VT patients and 1228 healthy subjects (*in silico *association) [19]. As indicated in Table 6, only two SNPs, *VPS8 *rs4686760 and *ACCN1 *rs12941510, showed some trend of association consistent with our hypothesis. The rs4686760-G allele found associated with decreased vWF levels was slightly less frequent in VT patients than in controls (0.441 vs 0.475, *P *= 0.101) and the rs12941510-A allele, associated with decreased FVIII levels, was also less frequent in cases than in controls (0.310 vs 0.350, *P *= 0.046). These associations can only be considered as suggestive as they would not pass correction for multiple testing. Nevertheless, the observed homogeneity of the allele frequencies of these two SNPs across all genotyped patients is noteworthy. Combining all the VT patients (n = 1946), and comparing to the healthy controls of the *in silico *GWAS, the association of rs4686760 with VT remained (0.454 vs 0.475, *P *= 0.108), and that of rs12941510 was strengthened (0.314 vs 0.348, *P *= 0.0056) (Table 7).

**Table 6:** In Silico Association With Venous Thrombosis of the Identified vWF- and FVIII Associated SNPs

		Alleles*	Minor Allele Frequency		Cochran Armitage *P*-value
			Cases	Controls	
vWF associated SNPs					
					
*VPS8*	rs4686760	A/G	0.441	0.475	*P *= 0.101
*EPB41L4A*	rs13361927	G/A	0.065	0.062	*P *= 0.797
*KRT18P24*	rs1634352†	G/A	0.284	0.318	*P *= 0.055
*12q21.33*	rs1438933	G/A	0.256	0.294	*P *= 0.051
*CDH2*	rs2298574	A/G	0.084	0.093	*P *= 0.444
*SAFB2*	rs732505	G/A	0.061	0.064	*P *= 0.713
					
FVIII associated SNPs					
					
*VAV2*	rs12344583	A/G	0.217	0.193	*P *= 0.133
*ACCN1*	rs1354492	G/A	0.476	0.469	*P *= 0.740
*ACCN1*	rs12941510	G/A	0.310	0.350	*P *= 0.046

*Common/minor alleles

† serves as proxy for rs1757948 (r^2 ^= 1).

No good proxy with r^2 ^> 0.80 was available for rs6708166 (*LBH*), rs1321761 (*FAM46A*) and rs7306642 (*S*TAB2)

**Table 7:** Genotype Distributions of rs4686760 and rs12941510 Across VT Samples.

	rs4686760			
	
	AA	AG	GG	MAF ^(2)^
MARTHA08	271	502	198	0.462
MARTHA10	173	281	115	0.449
GWAS patients	129	196	81	0.441
				
All VT patients	573	979	394	0.454
GWAS controls	354	581	292	0.475
	Test of association *P *= 0.108^(1)^			
	
	rs12941510			
	AA	AG	GG	MAF
MARTHA08	93	409	469	0.306
MARTHA10	67	243	259	0.331
GWAS patients	45	161	199	0.310
				
All VT patients	205	813	927	0.314
GWAS controls	139	576	512	0.348
	Test of association *P *= 0.0056			

^(1) ^Cochran Armitage trend test

^(2) ^Minor Allele Frequency

## Discussion

Theoretically, a sample size of 1,624 unrelated individuals should have a power of 95% to detect, at the significant level of 1.12 10^-7^, the additive allele effect of a SNP explaining at least 3% if the variability of a quantitative trait [28]. This power would decrease to 86% and 66% for a SNP explaining 2.5% and 2%, respectively. Our meta-analysis of 1,624 carefully selected samples did not reveal any genome-wide significant association suggesting that the additional common SNPs tagged by current GWAS array and influencing vWF and FVIII plasma levels left to be identified would, if any, individually explain less than 2% of the variability of these two traits.

By lowering the statistical stringency to p < 10^-5 ^but focusing on the homogeneity of the effects observed in three independent samples, we identified several novel candidate genes that could contribute to modulate the variability of vWF and FVIII, and that deserve to be further studied. The novel candidate genes for vWF are *VPS8*, *EBP41L4A*, *KRT18P24*, *SAFB2 *and a region on 12q21.3 where no known gene maps. Unfortunately, little is known about the biology of the associated proteins and their role in cardiovascular diseases. Among these, *VPS8 *stands out. The rs4686760-G allele of the *VPS8 *gene, which was associated with decreased vWF levels, was also observed less frequently in VT cases than in healthy controls (0.45 vs 0.48) in the *in silico *GWAS, although this observation did not reach significance (*P *= 0.10). The vacuolar protein sorting 8 homolog gene (*VPS8*) is involved in protein traffic between the golgic appartus and the vacuaole [29] and could participate to the regulation of urokinase-type plasminogen activator [30], the latter known to be involved in thrombosis.

For FVIII levels, the candidate genes identified in our study were *LBH*, *FAM46A*, *VAV2*, *S*TAB2 and *ACCN1*. Both *LBH *and *VAV2 *genes are thought to be involved in angiogenesis. The transcriptional cofactor limb-bud-and-heart (Lbh) was discovered as a small acidic nuclear protein highly conserved among species [31]. It has been demonstrated a dramatic suppression of VEGF mRNAs in cells that overexpress Lbh [32]. Vav2 is a guanine nucleotide exchange factor for Rho family proteins. The expression of a dominant negative form of Vav2 suppress the Vascular Endothelial-Protein Tyrosine Phosphatise (VE-PTP)-induced changes in endothelial cell morphology, such changes being implicated in regulation of angiogenesis [33].

Interestingly, we had previously shown that *S*TAB2 was located within a linkage peak for vWF levels in our FVL extended families [9] while almost concomitantly *S*TAB2 SNPs were found associated with both FVIII and vWF in the CHARGE consortium GWAS [16]. However, the non-synonymous rs7306642 (Pro2039Thr) found associated here with FVIII levels did not show a homogeneous effect on vWF levels across the three GWAS datasets (data not shown), and was in very low LD with others *S*TAB2 SNPs found associated with these plasma levels. The substitution of a Proline by a Threonine at position 2039 is predicted to be damaging according to web resources http://genetics.bwh.harvard.edu/pph/index.html; http://www.rostlab.org/services/SNAP. Investigating the effect of this substitution on VT risk would have been relevant but the corresponding SNP did not pass quality control in our *in silico *GWAS. These observations nevertheless suggest that an in-depth haplotype analysis of the *S*TAB2 gene are required to gain better insight into which SNPs more likely influence plasma levels of FVIII and/or vWF.

*ACCN1*, encoding an amiloride-sensitive cation channel implicated in cell growth and migration [34], is another gene that deserves greater attention as its genetic variability was found here associated with both FVIII levels and VT risk. However, the SNP that seemed to modulate FVIII levels the most, rs1354492, was not the one that showed association with the disease. This could suggest that either different SNPs distinctly influence plasma levels and VT risk, or that the identified SNPs are in LD with unmeasured variant(s) that could simultaneously influence both phenotypes.

Our meta-analysis was also able to replicate several of the previously reported associations between SNPs and vWF/FVIII levels. Replicated associations include vWF-associated SNPs at *STXBP*5, *VWF*, *STX2*, *TC2N *and *CLEC4M *genes, and FVIII-associated SNPs within *SCARA5 *and *VWF *genes. Other previously reported associations were not replicated, such as those involving *LDLR*, *BAI3*, and *S*TAB2 SNPs [5,9,16]. In addition to a lack of power, as previously discussed, this could be due to differential effects of SNP in normal range of plasma levels compared to the higher levels observed in VT patients. This could apply to the association of *BAI3 *with vWF levels observed in healthy nuclear families [9] where plasma levels were lower than those observed in our VT samples. Conversely, this explanation does not completely hold for the *LDLR *SNPs that were found associated with FVIII activity in a population [5] where FVIII activity in healthy individuals were at higher levels than those observed in our VT patients. Besides, in these two studies, different methods from those we have used here were employed to measure vWF and FVIII activity, and this could also contribute to the discrepancies observed in our study.

## Conclusions

In conclusion, a carefully planned meta-analysis of three independent samples gathering 1,624 individuals genotyped for more than 400,000 SNPs all over the genome replicated very recent findings but did not reveal any new genetic factors that could individually explain at least 2% of the plasma variability of vWF and FVIII levels.

## Competing interests

The authors declare that they have no competing interests.

## Authors' contributions

PEM, ML, FG and DAT designed the study and directed its implementation. GA, TOM and AD carried out statistical analyses. MG and WC were responsible for data collection and database management. GA drafted the article that was further reviewed by PEM, FG and DAT.

All authors read and approved the final manuscript.

## Pre-publication history

The pre-publication history for this paper can be accessed here:

http://www.biomedcentral.com/1471-2350/12/102/prepub

## Supplementary Material

Additional file 1 **FVL Family Questionnaire**. Click here for file

Additional file 2 **MARTHA questionnaire**. Excel file illustrating the questionnaire used for selecting MARTHA VT patients. Click here for file

Additional file 3 **Figure S1. Genotype filtering strategy applied to the three GWAS datasets**. ^(1) ^A genotype calling rate of > 0.90 was used in the FVL families and a threshold of 0.99 was used for the MARTHA patients. ^(2) ^SNPs with minor allele frequency less than 0.04 and 0.01 in FVL families and MARTHA patients, respectively, were excluded from the analysis. ^(3) ^SNPs demonstrating deviation from Hardy-Weinberg equilibrium at p < 10^-5 ^were excluded. 217 SNPs failed the genotype calling criterion simultaneously in the three study samples and this number was 19,111 for the minor allele frequency criterion. 19 SNPs failed the Hardy-Weinberg criterion in MARTHA08 and MARTHA10. Click here for file

Additional file 4 **Table S1. Haplotype Association Analysis of *ACCN1 *rs1354492 and rs12941510 With Plasma FVIII levels in MARTHA08 and MARTHA10 Studies**. ^(1) ^Haplotypic effect associated with each haplotype by comparison to the most frequent AG haplotype under the assumption of haplotype additive effects. Analyses were adjusted for age, sex and *ABO *blood group. Click here for file
