# Supplementary material for: Combined analysis of three genome-wide association studies on vWF and FVIII plasma levels
Source: BMC Med Genet. 2011 Aug 2;12:102. doi: 10.1186/1471-2350-12-102 (PMC3163514; doi:10.1186/1471-2350-12-102)
Supplement: Additional file 1 — FVL Family Questionnaire. [file 1471-2350-12-102-S1.PDF]

CONFIDENTIAL PATIENT INFORMATION

1. **Date information Collected:** |__|__| / |__|__| / |__|__|

2. **Center:** |__|__|

3. **Patient's Name:** ______________________
Last Name *First Name* *Initial*

Address: ______________________
____
____

Telephone: ______________
Area Code

Hospital ID Number: ______________

4. **Family Physician:** ______________________
Last Name *First Name* *Initial*

Address: ______________________
____
____

Telephone: ______________
Area Code

5. **Date of Baseline Assessment:** |__|__| / |__|__| / |__|__|
yy mm dd

6. **Gender:** ☐ Male ☐ Female

7. **Date of Birth:** |__|__| / |__|__| / |__|__|
yy mm dd

8. **Height (objective measurement):** |__|__|__| Meters

9. **Weight (objective measurement):** |__|__|__| Kilograms

10. Ethnic origin:

- ☐ Asian/Pacific Islander
- ☐ African
- ☐ Latin American
- ☐ Northern European
- ☐ Southern European
- ☐ Aboriginal
- ☐ Middle Eastern
- ☐ Other (describe)________________

12. Smoking behaviour (cigarettes)

Current use:

- None ☐
- Daily < 10 cig./day ☐
- Heavy > 11 cig./day ☐

Lifetime use:

- Max. # of cig./day |__|__|__|
- Duration of smoking (yrs) |__|__|__|

13. Number of previous episodes of venous thromboembolism: |__|__|

14. Age at first venous thromboembolism: |__|__|

15. Number of previous episodes of idiopathic venous thromboembolism: |__|__|

16. Surgery within last 6 months: Yes ☐ No ☐

17. Cancer within last 6 months: Yes ☐ No ☐

18. Immobilized by illness for ≥ 3 days within the last 6 months: Yes ☐ No ☐

19. Immobilization by plaster cast or splint for ≥ 3 days within the last 6 months:

Yes ☐ No ☐

20. Family History (1st degree relatives) of objectively documented venous thromboembolism:

Yes ☐ No ☐

MEDICATION AFFECTING HAEMOSTATIC OR LIPID-RELATED TRAITS

1. Was the patient taking any of the following types of medication(s) on a regular basis in the year prior to the study?

NO	YES		
☐	☐	Antiplatelet agent:	*If YES, Specify generic name, dose, duration:* ________________
☐	☐	Hormonal replacement:	*If YES, Specify generic name, dose, duration:* ________
☐	☐	Oral contraceptives:	*If YES, Specify generic name, dose, duration:* ________________
☐	☐	Anticoagulants:	*If YES, Specify generic name, dose, duration:* ________________
☐	☐	Lipid-lowering agent:	*If YES, Specify generic name, dose, duration:* ________________
☐	☐	Diabetic treatment:	*If YES, Specify generic name, dose, duration:* ________
☐	☐	Hypertensive treatment:	*If YES, Specify generic name, dose, duration:* ________________
☐	☐	Nicotine treatment:	*If YES, Specify generic name, dose, duration:* ________________

MEDICAL CONDITION(S)

1. **Does the patient have a history of:**

			Onset
**Hypertension:**	Yes ☐	No ☐	|__|__| / |__|__| mm yy
**Diabetes:**	Yes ☐	No ☐	|__|__| / |__|__| mm yy
**Hyperlipidemia:**	Yes ☐	No ☐	|__|__| / |__|__| mm yy
**Cancer:**	Yes ☐	No ☐	|__|__| / |__|__| mm yy
	If yes, type:________________		
**Coronary Artery Disease:**	Yes ☐	No ☐	|__|__| / |__|__| mm yy
**Peipheral Vascular Disease:**	Yes ☐	No ☐	|__|__| / |__|__| mm yy
**Venous Thrombosis:**	Yes ☐	No ☐	|__|__| / |__|__| mm yy
**Stroke:**	Yes ☐	No ☐	|__|__| / |__|__| mm yy
**Previous MI**	Yes ☐	No ☐	|__|__| / |__|__| mm yy

VENOUS THROMBOSIS HISTORY

1. **Patient has had DVT:** Yes ☐ No ☐

Diagnosed by:

Ultrasonography ☐ Venography ☐

2. **Patient has had PE:** Yes ☐ No ☐

Diagnosed by:

Ventilation / Perfusion Lung Scan ☐ Pulmonary Angiography ☐ Spiral CT ☐

3. **Most recent DVT/PE was idiopathic:** Yes ☐ No ☐

Most recent DVT/PE was due to transient risk factor: Yes ☐ No ☐

Most recent DVT/PE was due to cancer: Yes ☐ No ☐

HYPERCOAGULABLE BLOOD WORK-UP

1. **APRC Level:**

☐ Not tested Date tested |__|__| / |__|__| / |__|__| (yy mm dd) ☐ Normal ☐ Abnormal
Result: |__| . |__|__| U/mL

If Abnormal:
Was the abnormality confirmed by repeat testing? **No** ☐ **Yes** ☐ ☐ Not Done

2. **APTT:**

☐ Not tested Date tested |__|__| / |__|__| / |__|__| (yy mm dd) ☐ Normal ☐ Abnormal
Result: |__ |__|__| seconds

If Abnormal:
Was the abnormality confirmed by repeat testing? **No** ☐ **Yes** ☐ ☐ Not Done

3. **FV**

☐ Not tested Date tested |__|__| / |__|__| / |__|__| (yy mm dd) ☐ Normal ☐ Abnormal
Result: |__| . |__|__| U/mL

If Abnormal:
Was the abnormality confirmed by repeat testing? **No** ☐ **Yes** ☐ ☐ Not Done

4. **FVII**

☐ Not tested Date tested |__|__| / |__|__| / |__|__| (yy mm dd) ☐ Normal ☐ Abnormal
Result: |__| . |__|__| U/mL

If Abnormal:
Was the abnormality confirmed by repeat testing? **No** ☐ **Yes** ☐ ☐ Not Done

5. **FVIII**

☐ Not tested Date tested |__|__| / |__|__| / |__|__| ☐ Normal ☐ Abnormal
yy mm dd Result: |__| . |__|__| U/mL

If Abnormal:
Was the abnormality confirmed by repeat testing? **No** ☐ **Yes** ☐ ☐ Not Done

6. **FIX**

☐ Not tested Date tested |__|__| / |__|__| / |__|__| ☐ Normal ☐ Abnormal
yy mm dd Result: |__| . |__|__| U/mL

If Abnormal:
Was the abnormality confirmed by repeat testing? **No** ☐ **Yes** ☐ ☐ Not Done

7. **FX**

☐ Not tested Date tested |__|__| / |__|__| / |__|__| ☐ Normal ☐ Abnormal
yy mm dd Result: |__| . |__|__| U/mL

If Abnormal:
Was the abnormality confirmed by repeat testing? **No** ☐ **Yes** ☐ ☐ Not Done

8. **FXII**

☐ Not tested Date tested |__|__| / |__|__| / |__|__| ☐ Normal ☐ Abnormal
yy mm dd Result: |__| . |__|__| U/mL

If Abnormal:
Was the abnormality confirmed by repeat testing? **No** ☐ **Yes** ☐ ☐ Not Done

9. **Fibrinogen**

☐ Not tested Date tested |__|__| / |__|__| / |__|__| ☐ Normal ☐ Abnormal
yy mm dd Result: |__| . |__|__| U/mL

If Abnormal:
Was the abnormality confirmed by repeat testing? **No** ☐ **Yes** ☐ ☐ Not Done

10. **PAI-1**

☐ Not tested Date tested |__|__| / |__|__| / |__|__| ☐ Normal ☐ Abnormal
yy mm dd Result: |__| . |__|__| U/mL

If Abnormal:
Was the abnormality confirmed by repeat testing? **No** ☐ **Yes** ☐ ☐ Not Done

11. PT(INR)

☐ Not tested Date tested |__|__| / |__|__| / |__|__| ☐ Normal ☐ Abnormal
yy mm dd
Result: |__| . |__|__|

If Abnormal:
Was the abnormality confirmed by repeat testing? **No** ☐ **Yes** ☐ ☐ Not Done

12. TF

☐ Not tested Date tested |__|__| / |__|__| / |__|__| ☐ Normal ☐ Abnormal
yy mm dd
Result: |__| . |__|__| U/mL

If Abnormal:
Was the abnormality confirmed by repeat testing? **No** ☐ **Yes** ☐ ☐ Not Done

13. TFPI (LACI)

☐ Not tested Date tested |__|__| / |__|__| / |__|__| ☐ Normal ☐ Abnormal
yy mm dd
Result: |__| . |__|__| U/mL

If Abnormal:
Was the abnormality confirmed by repeat testing? **No** ☐ **Yes** ☐ ☐ Not Done

14. TPA

☐ Not tested Date tested |__|__| / |__|__| / |__|__| ☐ Normal ☐ Abnormal
yy mm dd
Result: |__| . |__|__| U/mL

If Abnormal:
Was the abnormality confirmed by repeat testing? **No** ☐ **Yes** ☐ ☐ Not Done

LIPID-RELATED BLOOD WORK-UP

1. ApoB

☐ Not tested   Date tested |__|__| / |__|__| / |__|__| ☐ Normal   ☐ Abnormal
yy mm dd   Result: |__| . |__|__| U/mL

If Abnormal:
Was the abnormality confirmed by repeat testing?   **No** ☐   **Yes** ☐   ☐ Not Done

2. HDL

☐ Not tested   Date tested |__|__| / |__|__| / |__|__| ☐ Normal   ☐ Abnormal
yy mm dd   Result: |__| . |__|__| U/mL

If Abnormal:
Was the abnormality confirmed by repeat testing?   **No** ☐   **Yes** ☐   ☐ Not Done

3. LDL

☐ Not tested   Date tested |__|__| / |__|__| / |__|__| ☐ Normal   ☐ Abnormal
yy mm dd   Result: |__| . |__|__| U/mL

If Abnormal:
Was the abnormality confirmed by repeat testing?   **No** ☐   **Yes** ☐   ☐ Not Done

Is a first degree relative known to have **factor VIII deficiency**?   ☐   ☐   ☐ Unknown

4. Total cholesterol

☐ Not tested   Date tested |__|__| / |__|__| / |__|__| ☐ Normal   ☐ Abnormal
yy mm dd   Result: |__| . |__|__| U/mL

If Abnormal:
Was the abnormality confirmed by repeat testing?   **No** ☐   **Yes** ☐   ☐ Not Done

5. Triglycerides

☐ Not tested   Date tested |__|__| / |__|__| / |__|__| ☐ Normal   ☐ Abnormal
yy mm dd   Result: |__| . |__|__| U/mL

If Abnormal:
Was the abnormality confirmed by repeat testing?   **No** ☐   **Yes** ☐   ☐ Not Done

6. **VLDL**

☐ Not tested Date tested |__|__| / |__|__| / |__|__| ☐ Normal ☐ Abnormal
yy mm dd Result: |__| . |__|__| U/mL

If Abnormal:
Was the abnormality confirmed by repeat testing? **No** ☐ **Yes** ☐ ☐ Not Done

7. **Lipoprotein a**

☐ Not tested Date tested |__|__| / |__|__| / |__|__| ☐ Normal ☐ Abnormal
yy mm dd Result: |__| . |__|__| U/mL

If Abnormal:
Was the abnormality confirmed by repeat testing? **No** ☐ **Yes** ☐ ☐ Not Done
