# Supplementary figures and images for: Combined analysis of three genome-wide association studies on vWF and FVIII plasma levels
Source: BMC Med Genet. 2011 Aug 2;12:102. doi: 10.1186/1471-2350-12-102 (PMC3163514; doi:10.1186/1471-2350-12-102)

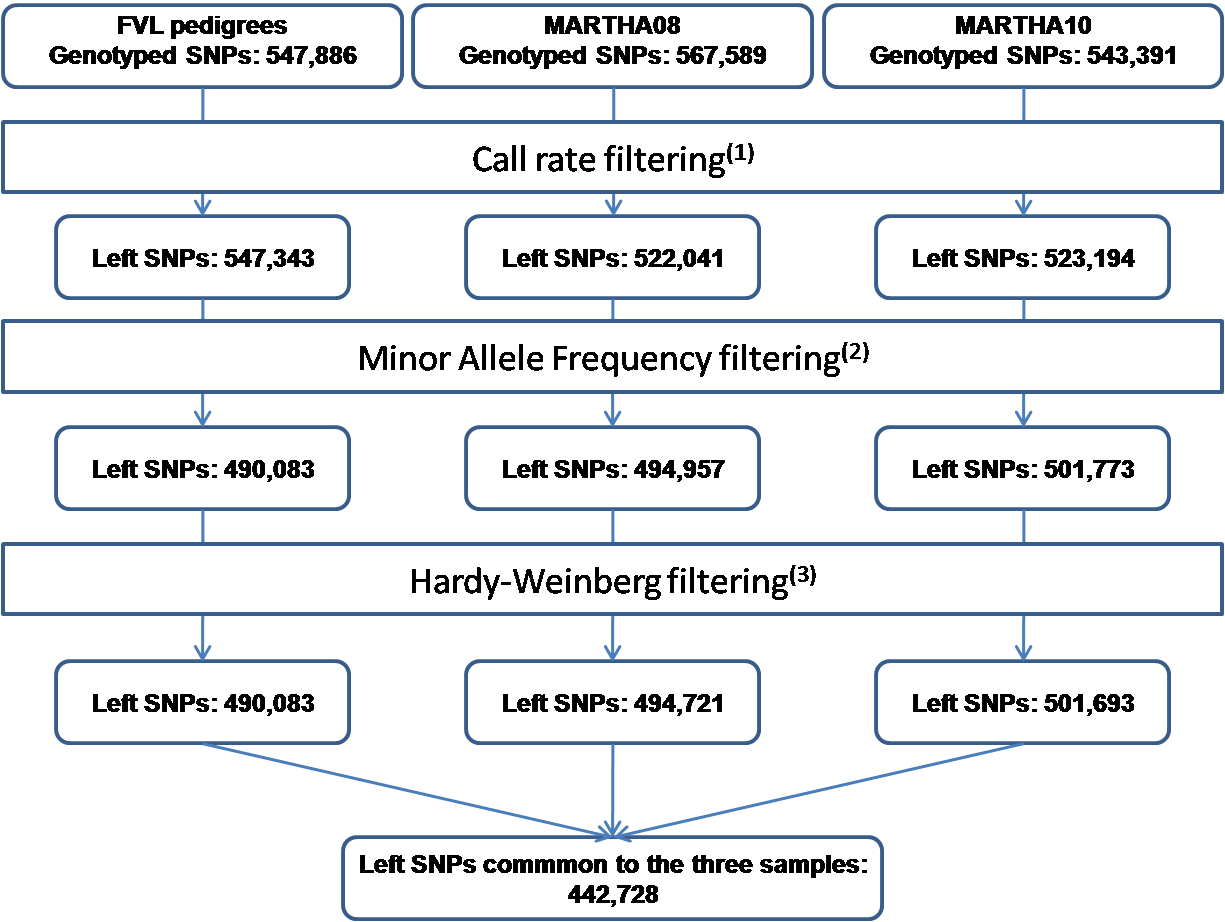

Supplement: Additional file 3 — Figure S1. Genotype filtering strategy applied to the three GWAS datasets. (1) A genotype calling rate of > 0.90 was used in the FVL families and a threshold of 0.99 was used for the MARTHA patients. (2) SNPs with minor allele frequency less than 0.04 and 0.01 in FVL families and MARTHA patients, respectively, were excluded from the analysis. (3) SNPs demonstrating deviation from Hardy-Weinberg equilibrium at p < 10-5 were excluded. 217 SNPs failed the genotype calling criterion simultaneously in the three study samples and this number was 19,111 for the minor allele frequency criterion. 19 SNPs failed the Hardy-Weinberg criterion in MARTHA08 and MARTHA10. [file 1471-2350-12-102-S3.TIFF]
