# Supplementary material for: Combined analysis of three genome-wide association studies on vWF and FVIII plasma levels
Source: BMC Med Genet. 2011 Aug 2;12:102. doi: 10.1186/1471-2350-12-102 (PMC3163514; doi:10.1186/1471-2350-12-102)
Supplement: Additional file 4 — Table S1. Haplotype Association Analysis of ACCN1 rs1354492 and rs12941510 With Plasma FVIII levels in MARTHA08 and MARTHA10 Studies. (1) Haplotypic effect associated with each haplotype by comparison to the most frequent AG haplotype under the assumption of haplotype additive effects. Analyses were adjusted for age, sex and ABO blood group. [file 1471-2350-12-102-S4.DOC]

**Haplotype Association Analysis of *ACCN1* rs1354492 and rs12941510 With Plasma FVIII levels in MARTHA08 and MARTHA10 Studies**

| rs1354492 | rs12941510 | MARTHA08 | | MARTHA10 | |
| --- | --- | --- | --- | --- | --- |
|  |  | Haplotype Frequencies | Haplotype effects^(1)^ 95% Confidence Interval | Haplotype Frequencies | Haplotype effects 95% Confidence Interval |
| G | G | 0.201 | -0.218 -0.356 , -0.079 | 0.196 | -0.070 -0.206 , -0.065 |
| G | A | 0.311 | -0.240 -0.340 , -0.120 | 0.330 | -0.141 -0.260 , -0.022 |
| A | G | 0.488 | reference | 0.474 | reference |

^(1)^ Haplotypic effect associated with each haplotype by comparison to the most frequent AG haplotype under the assumption of haplotype additive effects. Analyses were adjusted for age, sex and *ABO* blood group.
